# Gedunin inhibits pancreatic cancer by altering sonic hedgehog signaling pathway

**DOI:** 10.18632/oncotarget.8055

**Published:** 2016-03-14

**Authors:** Ramadevi Subramani, Elizabeth Gonzalez, Sushmita Bose Nandy, Arunkumar Arumugam, Fernando Camacho, Joshua Medel, Damilola Alabi, Rajkumar Lakshmanaswamy

**Affiliations:** ^1^ Center of Emphasis in Cancer Research, Department of Biomedical Sciences, Texas Tech University Health Sciences Center, Paul L. Foster School of Medicine, El Paso, Texas-79905, USA; ^2^ Graduate School of Biomedical Sciences, Texas Tech University Health Sciences Center, El Paso, Texas-79905, USA

**Keywords:** pancreatic cancer, gedunin, metastasis, hedgehog/gli signaling, apoptosis

## Abstract

**INTRODUCTION:**

The lack of efficient treatment options for pancreatic cancer highlights the critical need for the development of novel and effective chemotherapeutic agents. The medicinal properties found in plants have been used to treat many different illnesses including cancers. This study focuses on the anticancer effects of gedunin, a natural compound isolated from *Azadirachta indica*.

**METHODS:**

Anti–proliferative effect of gedunin on pancreatic cancer cells was assessed using MTS assay. We used matrigel invasion assay, scratch assay, and soft agar colony formation assay to measure the anti–metastatic potential of gedunin. Immunoblotting was performed to analyze the effect of gedunin on the expression of key proteins involved in pancreatic cancer growth and metastasis. Gedunin induced apoptosis was measured using flow cytometric analysis. To further validate, xenograft studies with HPAC cells were performed.

**RESULTS:**

Gedunin treatment is highly effective in inducing death of pancreatic cancer cells via intrinsic and extrinsic mediated apoptosis. Our data further indicates that gedunin inhibited metastasis of pancreatic cancer cells by decreasing their EMT, invasive, migratory and colony formation capabilities. Gedunin treatment also inhibited sonic hedgehog signaling pathways. Further, experiments with recombinant sonic hedgehog protein and Gli inhibitor (Gant-61) demonstrated that gedunin induces its anti–metastatic effect through inhibition of sonic hedgehog signaling. The anti–cancer effect of gedunin was further validated using xenograft mouse model.

**CONCLUSION:**

Overall, our data suggests that gedunin could serve as a potent anticancer agent against pancreatic cancers.

## INTRODUCTION

Pancreatic ductal adenocarcinoma remains a highly malignant disease with very poor prognosis. It is the 4^th^ leading cause of cancer related deaths in the United States and it is projected to become the 2^nd^ leading cause of cancer related deaths by the year 2030 [1–3]. Mortality rates are the highest with a 5-year survival rate of approximately 6% for all stages of pancreatic cancer [4]. It is estimated that 48,960 new cases will be diagnosed with pancreatic cancer in the U.S. and 337,000 globally by the end of 2015 [4]. The high metastatic nature and late stage diagnosis of this disease mainly contribute to its high lethality establishing an essential need for the development of novel chemotherapeutic agents against this deadly disease.

Many phytochemicals have been explored for their abilities to not only treat/prevent carcinogenesis progression but also for their abilities to overcome the extreme side effects of chemotherapy [1]. *Azadirachta indica*, commonly known as Neem, is a tree found predominantly in Asian and African countries where it has been widely used for its many medicinal properties [5]. Isolated Neem extracts have been studied for their anti–cancer effects on variety of cancers such as breast, colon, cervical, amongst others [6–10]. We have previously demonstrated the efficacy of ethanolic Neem leaf extracts in inhibiting mammary carcinogenesis by altering proliferation, apoptosis and angiogenesis [11]. Gedunin (tetranortriterpenoid) is an active ingredient derived from Neem extracts. Gedunin has shown to have potential anticancer activity by inhibiting proliferation of breast cancer cells via modulation of certain heat shock proteins [12]. Additionally, anti–proliferative effects have been observed in ovarian cancer cells in response to gedunin treatment through regulation of important signaling pathways [13].

Mutated KRAS is observed in >90% of pancreatic cancer cases and its downstream mediator, GLI1, is responsible for KRAS–induced pancreatic development/transformation [14]. Other studies demonstrated that GLI1 transcription factor acts synergistically with activated KRAS to induce metastatic pancreatic cancer [15]. Aberrant Hedgehog/GLI signaling pathway activity has been associated with growth and progression in many cancer types including pancreatic cancer [16]. It was discovered that sonic hedgehog (Shh) was aberrantly expressed in 70% of pancreatic adenocarcinoma tissues [17]. Overexpression of Shh alone was sufficient to induce pancreatic intraepithelial neoplasia (PanIN) [18]. Shh signaling activates GLI transcription factors which possess oncogenic characteristics and are deregulated in malignant cancers resulting in invasion and metastasis, chemo–resistance, and epithelial-to-mesenchymal transition [19]. According to previous reports, inhibition of upstream target sonic hedgehog (Shh) results in decreased expression of GLI1, therefore inhibiting tumor growth in xenograft models [17]. This highlights the crucial role of the Hedgehog/GLI signaling pathway in K-RAS driven pancreatic cancer for chemotherapeutic developments.

Herein, we demonstrate that gedunin induces anticancer activity on pancreatic cancer by inhibiting proliferation and metastasis through alteration of Shh/GLI1 signaling pathway and also by inducing apoptotic cell death.

## RESULTS

### Gedunin inhibits cell viability of pancreatic cancer cells

**Figure 1:**
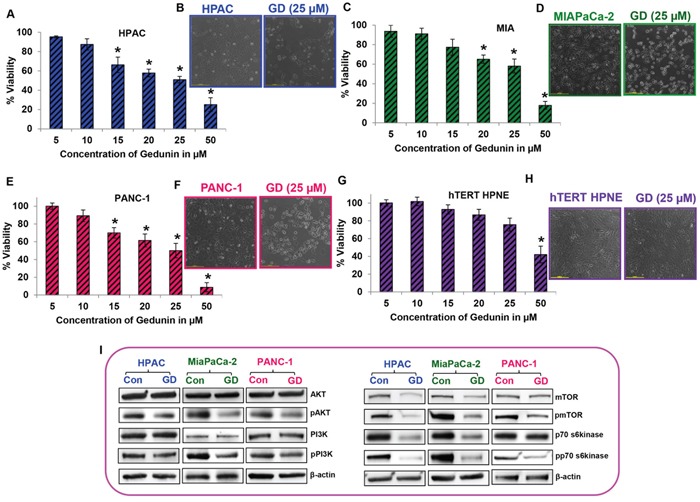
Gedunin inhibits cell viability of pancreatic cancer cells **A. C. E**. and **G**. Cells were treated with 5-50μM gedunin (GD). Cell viability was assessed using MTS analysis after treatment with different doses of gedunin for 24h. **B. D. F**. and **H**. Control and 25μM gedunin treated cells (their IC_50_ concentration) were visualized under light microscope at 10X magnification. **I**. Western blot analysis of total and phosphorylated forms of AKT, PI3K, mTOR, and p70s6kinase in pancreatic cancer cell lysates treated with 25μM gedunin. Data are expressed as the mean ± SEM (*p<0.05) of three separate experiments.

The effect of gedunin on cell viability of pancreatic cancer cell lines was tested using an MTS assay. Upon 24h of gedunin treatment, a dose–dependent decrease in cell viability was observed in HPAC, MIAPaCa-2, and PANC-1 cells with increasing concentrations of gedunin (5 to 50 μM). The cell viability analysis exhibited that gedunin induced approximately 50% cell death at 25μM concentration in all three pancreatic cancer cell lines (Figure 1A-1F). However, 25μM of gedunin was not able to significantly alter cell viability of normal pancreatic cells (hTERT-HPNE) (Figure 1G) suggesting that gedunin selectively kills pancreatic cancer cells and is nontoxic to normal pancreatic cells. In addition, microscopic analysis also showed no or minimal reduction in cell number of normal pancreatic cells (hTERT-HPNE) treated with gedunin (Figure 1H). This data is encouraging because gedunin seems to be specifically targeting pancreatic cancer cells more efficiently than normal pancreatic cells. Proliferative markers such as phosphorylated forms of AKT, PI3K, mTOR, and p70S6K were significantly down–regulated in the pancreatic cancer cell lines treated with 25μM gedunin (Figure 1I). These results demonstrate the anti–proliferative potential of gedunin on aggressive pancreatic cancer cells

### Gedunin induces apoptosis in pancreatic cancer cells

**Figure 2:**
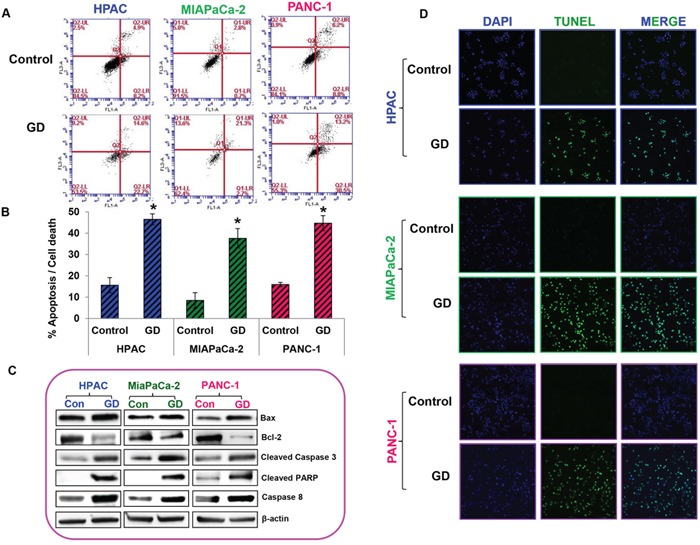
Gedunin induced apoptosis in pancreatic cancer cells **A**. and **B**. Apoptosis of pancreatic cancer cells treated with 25μM gedunin was detected using flow cytometry. After 24h treatment with Gedunin, cells were stained with Annexin V-FITC/PI. HPAC, MIAPaCa-2, and PANC-1 cells were treated with 25μM gedunin for 24h and protein lysates were subjected to immunoblotting. **C**. Western blotting was performed using antibodies against Bax, Bcl-2, Cleaved Caspase 3, Cleaved PARP, and Caspase 8. **D**. Pancreatic cancer cells were treated with 25μM gedunin for 24h and then labeled with DAPI and TUNEL solution. Detection of TUNEL positive cells was observed using confocal microscopy. Data are expressed as the mean ± SEM (*p<0.05) of three separate experiments.

The effect of gedunin on apoptosis was analyzed using Annexin V–FITC Apoptosis Detection Kit I. Gedunin treatment (25μM) demonstrated the induction of apoptosis in HPAC (46.5%), MIAPaCa-2 (37.6%), and PANC-1 (44.7%) cells (Figures 2A and 2B). Pro–apoptotic markers Bax, cleaved Caspase 3, and cleaved PARP were increased in response to gedunin treatment in all three pancreatic cancer cells. Interestingly, Bcl-2, an anti–apoptotic marker, was decreased in gedunin–treated pancreatic cancer cells. In addition, Caspase 8, one of the signature markers for death receptor pathway of apoptosis was increased in response to gedunin treatment. This indicates that gedunin–treatment induces both intrinsic and extrinsic mediated apoptotic cell death (Figure 2C). TUNEL assay results further confirmed the apoptotic effect of gedunin on pancreatic cancer cells (Figure 2D).

### Gedunin reduces the migratory characteristics of pancreatic cancer cells

**Figure 3:**
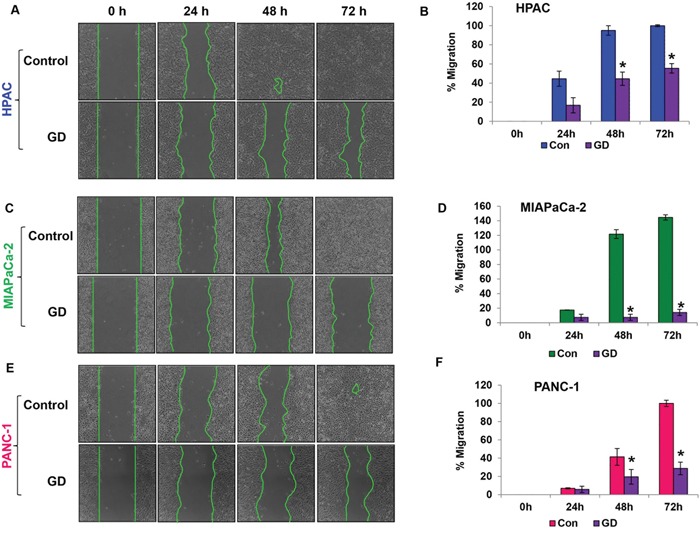
Gedunin reduces the migratory characteristics of pancreatic cancer cells **A. C**. and **E**. The migratory capabilities of HPAC, MIAPaCa-2, and PANC-1 cells treated with 15μM gedunin were evaluated using the scratch assay. **B. D**. and **F**. Graphical representation of migration in pancreatic cancer cells was calculated via NIS-Element AR software. Data are expressed as the mean ± SEM (*p<0.05) of three separate experiments.

The migratory ability of a cancer cell is crucial for the development of metastasis. The effect of gedunin on HPAC, MIAPaCa-2 and PANC-1 cells migratory capabilities was evaluated using the scratch assay. Gedunin treatment (15μM) effectively inhibited the migratory abilities of all pancreatic cancer cells compared to control groups. The control cells migrated and completely filled the scratch within 72h. Even after 72h, HPAC, MIAPaCa-2, and PANC-1 cells treated with gedunin were only able to migrate ~55%, 14%, and 28%, respectively (Figure 3A-3F).

### Metastasis is reduced in gedunin treated pancreatic cancer cells

**Figure 4:**
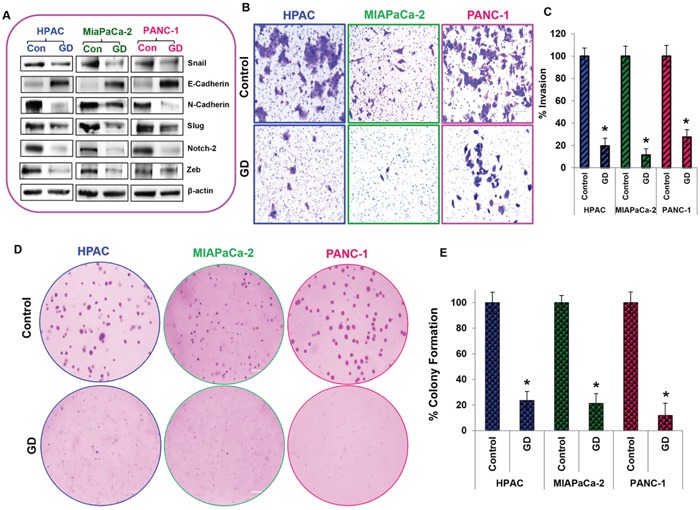
Metastasis is reduced in gedunin treated pancreatic cancer cells **A**. Expression levels of EMT signaling proteins (Snail, E-Cadherin, N-Cadherin, Vimentin, Slug, Notch 2, and Zeb) were measured by Western blotting. **B**. and **C**. Matrigel invasion assay evaluating invading abilities of pancreatic cancer cells treated with 15μM gedunin, which was recorded using Nikon Eclipse TS 100 microscope (20X magnification). **D**. and **E**. Soft agar assay represents inhibition of colony formation in HPAC, MIAPaCa-2 and PANC-1 cells treated with 15μM gedunin. Data are expressed as the mean ± SEM (*p<0.05) of three separate experiments.

Epithelial to mesenchymal transition, invasion and colony forming capacities are important events involved in metastatic progression. Using Western blots we studied the expression of key proteins involved in the EMT process, which drives the metastatic progression of cancers. We observed that pro–EMT markers such as Snail, N-cadherin, Vimentin, Slug, Notch 1 & 2, and Zeb were downregulated while anti–EMT marker, E-cadherin, was upregulated with 25μM gedunin treatment (Figure 4A). Next, we used the Matrigel–coated Boyden chambers to assess the effect of gedunin on the invasive capabilities of the pancreatic cancer cells. Treatment with 15μM of gedunin profoundly inhibited the invasive capacity of HPAC, MIAPaCa-2 and PANC-1 cells by 80.5%, 88.6%, and 72.5% respectively (Figures 4B and 4C). Further, soft agar assays were conducted to observe the effects of gedunin on colony forming abilities of pancreatic cancer cell lines. Crystal violet staining showed the reduced size and number of colonies in gedunin treated cells compared to the control groups (Figure 4D). Quantitative analysis revealed that gedunin inhibited ~80 to 88% of colony forming ability of pancreatic cancer cells (Figure 4E). These results indicate that gedunin is effective in blocking EMT.

### Sonic hedgehog/Gli–signaling was reduced upon gedunin treatment in pancreatic cancer cells

**Figure 5:**
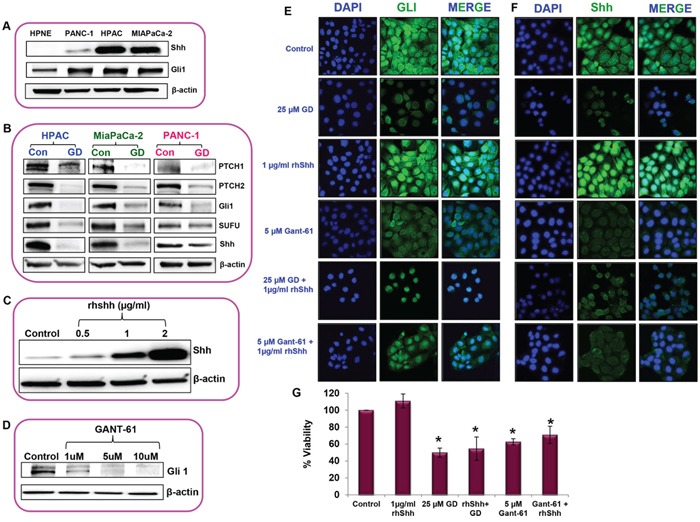
Sonic hedgehog/Gli–signaling was reduced upon gedunin treatment in pancreatic cancer cells **A**. The expression levels of Shh and Gli1 in normal pancreatic cells (hTERT-HPNE) and pancreatic cancer cell lines (PANC-1, HPAC and MIAPaCa-2). **B**. Western blot analysis of Hedgehog/Gli1 signaling protein expressions in pancreatic cancer cells treated with 25μM gedunin. **C**. and **D**. Standardization analysis to identify the effective dose of rhShh and Gli1 inhibitor (GANT-61) in HPAC cells using Western blot analysis. Immunofluorescence analysis of Gli1 **E**. and Shh **F**. expression in HPAC cells treated with 25μM gedunin, 1μg/ml rhShh with or without gedunin, and 5μM GANT-61 with or without 1μg/ml rhShh. Immunofluorescence images were captured using confocal microscopy at 60X magnification. Experiments were done in triplicates. **G**. Cell viability of HPAC cells were studied using MTS assay on cells were treated with 1μg/ml rhShh, 25μM gedunin alone or in combination with 1μg/ml rhShh, and 5 μM GANT-61 alone or in combination with 1μg/ml rhShh. Data are expressed as the mean ± SEM (*p<0.05) of three separate experiments.

The Sonic hedgehog/Gli signaling pathway has been shown to promote cancer progression. Shh and Gli1 were upregulated in all pancreatic cancer cell lines such as HPAC, MIAPaCa-2, and PANC-1 compared to normal pancreatic cell line hTERT HPNE (Figure 5A). To understand the molecular mechanism behind the anti–cancer effects of gedunin on pancreatic cancer, we sought to investigate the role of gedunin on Hedgehog signaling pathway. Gedunin treated pancreatic cancer cells were effectively downregulated PTCH1, PTCH2, Gli1, SUFU and Shh proteins involved in Hedgehog/Gli signaling pathway (Figure 5B). HPAC cells were used for further experiments due to their aggressive nature and higher protein expression levels of Shh.

Exogenous supplementation of rhShh (0.5, 1, and 2μg/ml) increased the expression levels of Shh in a dose–dependent manner, compared to the control cells (Figure 5C). Based on our findings we decided to use 1μg/ml rhShh for further experiments as it was sufficient enough to significantly increase the expression of Shh levels similar to highest dose tested (2μg/ml rhShh) in comparison to the control cells. Then GANT-61 a Gli inhibitor was used to investigate the effect of Gli in gedunin–induced anticancer effect. GANT-61 significantly inhibited the Gli1 expression in a dose–dependent manner. Our results showed that 5μM GANT-61 was as effective as the highest dose tested (10μM), so we chose 5μM dose for further studies (Figure 5D). Immunofluorescence microscopy provided further insight on the level of intracellular Gli1 and Shh expression in response to different treatments. Cells treated with 1μg/ml rhShh demonstrated higher expression levels of Gli1 and Shh in comparison to the control cells. Whereas HPAC cells treated with 25μM gedunin or 5μM GANT-61 had minimal expression levels of Gli1 and Shh. Interestingly, the addition of gedunin to rhShh treatment group resulted in a significant reduction in Gli1 and Shh expression. Inhibition of Gli1 through GANT-61 decreased the expression levels of Shh indicating the possibility of a feedback regulation in Hedgehog signaling by Gli1 (Figures 5E and 5F). These data demonstrate gedunin's ability to negatively influence the Hedgehog/Gli pathway.

Our results further demonstrate that overexpression of Shh resulted in marginally increasing cell viability. Gedunin, however was able to inhibit cell viability even in cells with high levels of Shh. In addition, treatment with Gli inhibitor GANT-61 also resulted in reduction in cell viability in pancreatic cancer cells overexpressing Shh (Figure 5G). These data indicate the gedunin inhibits pancreatic cancer cell viability by targeting the Shh/Gli signaling pathway.

### Gedunin inhibits metastasis of pancreatic cancer cell through the sonic hedgehog/Gli signaling

**Figure 6:**
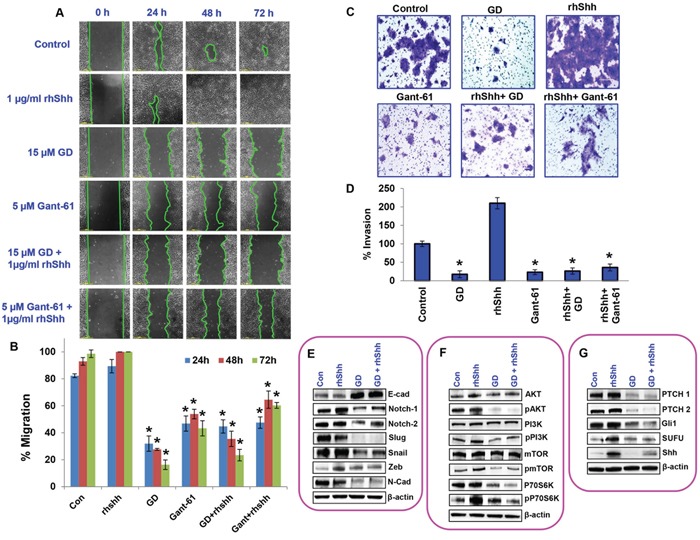
Gedunin inhibits growth and metastasis of pancreatic cancer cell through the sonic hedgehog/Gli signaling **A**. Migratory abilities of HPAC cells treated with 15μM gedunin, 1μg/ml rhShh with or without 15μM gedunin, and 5μM GANT-61 with or without 1μg/ml rhShh were recorded for 72h using Nikon Biostation CT. **B**. Quantification of distance migrated by the cells was obtained via NIS-Element AR software. **C**. and **D**. The invading capabilities of HPAC cells with various treatments (15μM gedunin, 1μg/ml rhShh with or without 15μM gedunin, and 5μM GANT-61 with or without 1μg/ml rhShh) were visualized using Nikon Eclipse TS 100 microscope (20 X magnifications) and invaded cells were also quantified from 5 different fields per treatment. Western blotting analysis of HPAC cells treated with 25μM gedunin or 1μg/ml rhShh alone or in combination was done using antibodies against **E**. EMT molecular markers, **F**. proliferative markers, and **G**. Hedgehog/Gli1 signaling markers. Data are expressed as the mean ± SEM (*p<0.05) of three separate experiments.

To further confirm gedunin's anti–metastatic characteristics, we tested events crucial for metastatic ailments by altering Shh/Gli signaling. Classic scratch assay was performed to assess the migratory capacity of cells. Cells treated with 1μg/ml rhShh migrated 100% of the scratch within 48h, while gedunin–treated (15μM) cells failed to close the scratch even after 72h. Cells treated with both 15μM gedunin and 1μg/ml rhShh had migrated only 23% of the scratch after 72h incubation, indicating gedunin's efficacy in halting migration even in the presence of exogenous rhShh (Figures 6A and 6B).

As expected, in matrigel invasion assay, cells treated with rhShh (1μg/ml) displayed exponentially higher numbers of invading cells, compared to the control group. Gedunin displayed reduced number of invaded cells, similar to GANT-61 (5μM). Addition of gedunin efficiently inhibited rhShh–induced invasion of pancreatic cancer cells. Our data demonstrates that Shh/Gli signaling is important for metastasis and that gedunin effectively targets this pathway resulting in reduced metastatic potential of pancreatic cancer cells (Figures 6C and 6D).

We further investigated the effect of gedunin on HPAC cells treated with rhShh in order to test its efficacy in inhibiting pancreatic cancer growth and metastasis. Our results demonstrate a significant downregulation of Notch 1 & 2, Slug, Snail, Zeb and N-Cadherin in response to gedunin treatment. Additionally, gedunin upregulated the expression of E-cadherin even in the presence of high levels of Shh (Figure 6E). Moreover, gedunin was highly effective in reducing the expression of proliferative markers such as pAKT, pPI3K, pmTOR, and pP70S6K, which were upregulated by rhShh treatment (Figure 6F). Gedunin similarly influenced the hedgehog signaling markers (PTCH1, PTCH2, Gli1, SUFU and Shh) (Figure 6G). All these data demonstrate that Shh/Gli signaling is enhanced in pancreatic cancer cells and gedunin effectively targets this signaling pathway and inhibits pancreatic cancer progression.

### Gedunin inhibited pancreatic cancer growth and metastasis in HPAC xenograft models

**Figure 7:**
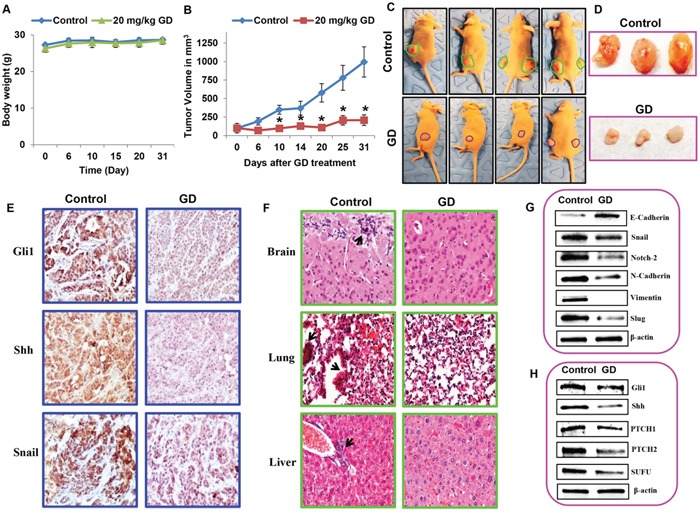
Gedunin inhibited pancreatic cancer growth and metastasis in HPAC xenograft models Nude mice bearing HPAC tumors (~100 mm^3^) were divided into two groups (i) Control (vehicle-DMSO) and (ii) gedunin (20 mg/kg body weight). **A**. Body weight and **B**. tumor volume were measured twice weekly for 30 days. **C**. Control and gedunin treated mice with HPAC xenografts. **D**. Representative data demonstrating excised tumors of both control and gedunin treated mice. **E**. Gedunin treated HPAC xenograft tissues were evaluated by IHC for Gli1, Shh, and Snail expression. **F**. Micrometastasis in control xenografts observed in brain, lung, and liver using hematoxylin and eosin staining. Immunoblots of xenograft tumor tissues for **G**. EMT markers and **H**. Hedgehog/Gli1 signaling markers. Data are expressed as the mean ± SEM (*p<0.05) of three separate experiments.

To further validate the anti–cancer activity of gedunin, we performed xenograft studies using athymic nude mice. HPAC cells (1 × 10^6^ cells) were transplanted subcutaneously in the flanks of the nude mice. After palpable tumors developed (~100 mm^3^), the nude mice were randomly divided into two groups (control and gedunin (20 mg/kg body weight) treatment groups, n=6). Gedunin was administered five times a week for a month, while the controls received vehicle treatment at the same frequency for the same duration. No significant difference in body weight was recorded for either group, indicating that gedunin treatment is safe and does not have any toxic effects (Figure 7A). Our results demonstrated that gedunin effectively suppressed pancreatic tumor growth as evidenced by a significant decrease in tumor volume compared to the untreated control group (Figure 7B, 7C and 7D). Immunohistochemical staining of the tumor tissue demonstrated a significant decrease of Gli1, Shh and Snail expression in the gedunin treated mice compared to the control group (Figure 7E). In control nude mice, micro–metastasis was observed in brain, liver, and lung; Gedunin–treated mice showed no metastasis to distant organs as illustrated through hematoxylin and eosin staining (Figure 7F). To further confirm the anti–metastatic effect of gedunin observed *in vitro*, we conducted immunoblotting for key molecular players of EMT. Pro–EMT markers N-Cadherin, Vimentin, and Notch 2 were downregulated, while the epithelial marker E-Cadherin was up regulated in gedunin–treated tumors (Figure 7G). Further, expression levels of molecular markers involved in the Shh signaling pathway were also analyzed. Consistent with our *in vitro* findings, mice treated with gedunin had a decreased expression of PTCH1, PTCH 2, Gli1, SUFU, and Shh in comparison to the control group (Figure 7H). These results corroborate our *in vitro* findings that gedunin suppresses metastasis via Hedgehog/Gli1 signaling, leading to reduced tumor burden.

## DISCUSSION

Pancreatic cancer carries the worst prognosis due to its metastatic nature. Unfortunately, the available conventional treatments for pancreatic cancer remain ineffective as a result of its acquired chemo–resistance, thus emphasizing the need for the development of novel chemotherapeutic strategies [20]. Natural compounds are reported to have the capabilities of inhibiting proliferation and inducing apoptosis in cancer cells [21]. In fact, they have revolutionized the field of cancer therapy development as; more natural compounds are being tested in the preclinical and clinical trials [22]. Gedunin, a bioactive compound derived from the Neem tree, appears to be an effective natural chemotherapeutic agent due to its potent anticancer effects on many types of cancer such as oral, prostate, ovarian, and colon [12]. Gedunin is a limonoid and it has been shown to be an effective anticancer drug [13, 23]. Using liquid chromatography/mass spectrometry (LC/MS) the human plasma levels of limonoids were estimated [24, 25]. They found that increasing doses of limonin was associated with increased availability of limonins in the plasma (1.74 to 5.27 nmol/L). Further, the mean time to maximum concentration in the plasma was observed at 6 hours. All these data clearly demonstrate that the limonoid-gedunin is bioavailable. In our present study, we found that gedunin is an effective anticancer agent against the human pancreatic adenocarcinoma cells. Further, we also demonstrate that gedunin did not have any toxic effects on the normal pancreatic cells. These findings are interesting and suggest that gedunin could be a potential chemotherapeutic drug for pancreatic cancer.

It is well known that both the intrinsic and extrinsic pathways are involved in cellular apoptosis [26]. Bcl-2 family is mainly involved in regulating intrinsic pathway of apoptosis, which is mainly dependent on the expression of Bcl-2 and Bax. On the other hand, increased expression of cleaved caspase 8 indicates the activation of extrinsic pathway of apoptosis [27]. We observed increased expressions of Bax, cleaved Caspase 3, cleaved Caspase 8 and cleaved PARP and while Bcl-2 expression was decreased in gedunin treated pancreatic cancer cells. Collectively, these data demonstrate that gedunin induces apoptosis through both the intrinsic and extrinsic pathways in pancreatic cancer cells. Furthermore, gedunin also impacts the PI3K/AKT/mTOR pathway in such a way to inhibit pancreatic cancer growth. PI3K/AKT/mTOR pathway plays a significant role in the growth and survival of various cancer cells [28]. Gedunin effectively inhibited the activation of PI3K and its downstream signaling by dephosphorylating PI3K at Tyr458/Tyr199, AKT at Ser473, p70S6K at Thr389 and mTOR at Ser2448. These data demonstrate that gedunin is effective in negatively regulating PI3K downstream signaling at several levels leading to reduced survival of pancreatic cancer cells.

Gedunin was highly effective in inhibiting the aggressive and metastatic nature of the pancreatic cells which was evident from the reduced levels of migration, invasion and also decreased colony forming capabilities. Epithelial-to-mesenchymal transition (EMT) is a crucial process involved in initiation and progression of metastasis [29]. During EMT there is loss or reduction of epithelial characteristics and the gain of mesenchymal characteristics leading to the loss of cell-to-cell adhesion, promotion of invasion, and subsequently induction of metastasis [30]. EMT can be initiated by many different signaling pathways and there are key molecules in these pathways that regulate EMT [31]. Our data demonstrated that gedunin inhibits EMT by decreasing the expression of mesenchymal markers N-Cadherin, Slug, Snail, Vimentin, Notch 1 & 2, and Zeb while increasing the expression of epithelial marker E-cadherin. These effects of gedunin could be attributed to the inhibition of the three essential metastatic events namely migration, invasion, and colony formation in pancreatic cancer cells. Together, our data shows that gedunin is able to inhibit cell proliferation, increase apoptosis, while at the same time inhibiting the metastatic characteristics of pancreatic cancer cells.

The inhibition or suppression of EMT in pancreatic cancer cells is significant and understanding the molecular mechanism is crucial for making advances in treating pancreatic cancers. Recent findings have deemed that molecularly targeted therapies are the most promising cancer therapies [32]. Oncogenic KRAS mutations account for >95% of pancreatic cancer development [33]. In addition, its constitutive activity stimulates downstream effectors such as Hedgehog signaling [34]. It has been discovered that various cancers contain aberrant Hedgehog signaling activation which is responsible for 1/3 of cancer–related deaths; making this pathway an ideal target for chemotherapeutic development [35]. Overexpression of Gli1, a downstream transcription factor of the Hedgehog signaling pathway, is observed in many cancers and is suggested to play a role in the development and progression of metastatic diseases [36]. Here we show that gedunin effectively targets the Shh/Gli pathway in pancreatic cancer cells. Our study demonstrated a significant increase of Shh and Gli1 expression in all the pancreatic cancer cell lines tested, confirming aberrant Shh/Gli1 activity in pancreatic cancers. Importantly, gedunin effectively reduced the expression of key molecular markers involved in the Shh signaling pathway including Shh, Gli1, PTCH1 and 2, and SUFU. Earlier it was reported that pancreatic cancer tissue had higher expression of Shh, PTCH1 and Gli 1 mRNA compared to adjacent normal pancreatic tissue [37, 38]. Although the direct crosstalk between Gli1 and EMT is not completely elucidated, it is known that Gli1 exhibits a pro–tumorigenic effect on pancreatic cancer cells by altering the EMT process [39]. GANT-61, a well–established Gli1 inhibitor was used to confirm if gedunin–induced its effect through Gli1. Our findings suggest that gedunin–induced Gli1 inhibition led to inhibition of pancreatic cancer migration and invasion even in the presence of exogenous rhShh. Based on our data, we can speculate that gedunin's anticancer potential is driven through inhibition of EMT by targeting Gli1.

To further validate our *in vitro* findings we used the *in vivo* xenograft model. Gedunin efficiently suppressed tumor growth in *in vivo* xenograft models with insignificant changes to body weight; suggesting that gedunin could be a safe anticancer agent with minimal side effects. The animals that received gedunin did not exhibit any discomfort. This is important to measure in order to avoid harsh and severe side effects usually recorded in many of the conventional chemotherapies available today [40]. Furthermore, gedunin inhibited micro–metastasis in the brain, liver and lung when compared to control animals. Similar to our *in vitro* data, we also observed that gedunin suppressed the Hedgehog/Gli1 signaling pathway and EMT in the xenograft tumors as well. These data validates that gedunin induces anticancer/anti–metastatic effects by targeting Hedgehog/Gli1 signaling pathway. In conclusion, our findings highlight the potential of gedunin as a potent anticancer drug for pancreatic cancer. Further studies are required to understand the pharmacokinetics and pharmacodynamics of gedunin for it to be considered as an anticancer agent with high clinical translational potential.

## MATERIALS AND METHODS

### Ethics statement

All experiments performed were reviewed and approved by the Texas Tech University Health Sciences Center Institutional Biosafety Committee and Institutional Animal and Care and Use Committee.

### Cell lines and reagents

hTERT HPNE (human normal pancreatic epithelial cells) and HPAC, PANC-1 and MIAPaCa-2 (pancreatic cancer cell lines) were purchased from the American Type Culture Collection. The cell lines were characterized and authenticated by ATCC based on morphology, karyotyping and PCR assays. Cells were cultured at 37°C in a humidified atmosphere containing 5% CO_2_. Similar to our prior studies, the above mentioned cell lines were supplemented with the appropriate culture medias such as Dulbecco's modified Eagle's media, M3 base Media and RPMI-1640 [41].

Gedunin was acquired from Tocris Bioscience (Bristol, UK). MTS reagent [3-(4,5-dimethylthiazol-2-yl)-5-(3-carboxymethoxyphenyl)-2-(4-sulfophenyl)-2H-tetrazolium] and DeadEnd Fluorometric TUNEL system were purchased from Promega (Madison, WI, USA). BSA was purchased from Sigma-Aldrich Corporation (St Louis, MO, USA). BD Pharmingen Annexin V-FITC Apoptosis Detection Kit I was obtained from BD Biosciences (San Diego, CA, USA). GANT-61 and Recombinant Sonic Hedgehog/Shh (C24II) were purchased from R&D Systems (Minneapolis, MN). Mammalian protein extraction reagent (mPER) was acquired from Thermo Scientific (Rockford, IL, USA). All experimental procedures, reagents and chemicals used in this study were approved by the Texas Tech University Health Sciences Center Institutional Biosafety Committee.

The primary antibodies used for western blot analysis were: pAKT (sc-101629), AKT (sc-5298), Bcl-2 (sc-783) and Notch 1 (sc-23299) obtained from Santa Cruz Biotechnology (Santa Cruz, CA, USA); Notch 2 (4530P), Snail (3879), E-cadherin (3195), N-cadherin (4061), Zeb (3396), Vimentin (5741), Slug (9585), Bax (2772), cleaved caspase-3 (9661), cleaved PARP (9542), pPI3K (4228), PI3K (4292), pPTEN (9549), pmTOR (2974), mTOR (4517), p-p70s6kinase (9206), p70s6kinase (9202), Shh (2207), PTCH1 (2468), PTCH2 (2470), SUFU (2520) and Gli1(3538) were procured from Cell Signaling Technology, Boston, MA, USA; Caspase 8 (ab 25901) was purchased from Abcam (Cambridge, MA, USA) and β-actin from Sigma Aldrich (St. Louis, MO, USA). Appropriate anti–mouse and anti–rabbit secondary antibodies were purchased from Santa Cruz Biotechnology (Santa Cruz, CA, USA).

### MTS assay

MTS assay was conducted to analyze cell proliferation. Cells (hTERT HPNE, HPAC, MIAPaCa-2, and PANC-1) were seeded in 96 well plates at a density of 0.3 × 10^4^ cells/ well and maintained in an incubator containing 5% CO_2_ at 37°C for 24h. Various concentrations of gedunin (5, 10, 15, 20, 25, and 50μM), 1μg/ml of rhShh, and 5μM of GANT-61) were added to the cells and incubated for 24h. MTS was added and incubated for 4h then the optical density was measured at 490 nm using a microplate reader (CLARIOstar, BMG LABTECH, Cary, NC, USA).

### Scratch assay

Cell migration was evaluated using scratch assay as described in our previous study [41]. HPAC and MIAPaCa-2 cells were seeded on a 6 well plate at a density of 8 × 10^5^ cells/well and 6 × 10^5^ cells/well for PANC-1. Cells were cultured at 37°C in 5% CO_2_ until monolayer confluency was reached. A scratch was made with a sterile pipette tip and washed twice with PBS and fresh media was added. Cells were treated with 15μM gedunin or vehicle (Dimethyl Sulfoxide).

HPAC cells were further studied using 1μg/ml of rhShh, 15μM gedunin, 5μM GANT-61, and combinations such as 15μM gedunin with 1μg/ml rhShh and 5μM GANT-61 with 1μg/ml rhShh in order to understand the involvement of sonic hedgehog signaling in pancreatic cancer cell migration. Images were taken at 2h intervals for a time period of 72h at 37°C using the Biostation CT (Nikon Instruments Inc. Melville, NY, USA). The distance migrated by the cells was calculated using NIS-Element AR software (24809702).

### Invasion assay

The invasive capabilities of pancreatic cancer cells with gedunin were assessed using the invasion assay. Cells were plated at a density of 5 × 10^4^ cells/well for PANC-1 and 8 × 10^4^ cells/well for HPAC and MIAPaCa-2 in the upper chamber of 6.5 mm transwell with 8.0μm pore polycarbonate membrane inserts (Corning Incorporated, Corning, NY, USA) coated with matrigel (Company, Bedford, MA, USA). Cells were treated with 15μM gedunin for 48h. Growth media (600μl) containing 10% FBS was added in the lower chamber of the transwell as a chemo–attractant. Invaded cells in the lower chamber were fixed and stained with 0.2% crystal violet in 5% formalin. Randomly selected fields, with a magnification of 20X, were imaged and captured via Nikon Eclipse TS 100 microscope.

In another set of experiments 15μM gedunin, 1μg/ml rhShh, 5μM GANT-61, 1μg/ml rhShh and 15μM gedunin, and 1μg/ml rhShh and 5μM GANT-61 were administered to HPAC cells for 48h to investigate the role of sonic hedgehog on the invasive capacity of pancreatic cancer cells.

### Colony formation assay

Pancreatic cancer cells at densities of 2 × 10^4^ cells were seeded on 60-mm dishes with a top layer of 0.7% agarose and a bottom layer of 1% agar. Treatment groups were administered with 15 μM of gedunin every 3 days for up to 39 days for PANC-1 cells and 60 days for HPAC and MIAPaCa-2. Cells were fixed and stained with 0.2% crystal violet in 5% formalin solution. Colonies were counted manually and images were captured using Nikon SMZ 1500 at 4X magnification. Experiments were conducted in triplicates.

### Western blot analysis

Total proteins from cultured cells and xenograft tumor tissues were loaded and separated using SDS-PAGE and transferred onto PVDF membranes. Blocking of membranes was done using 5% bovine serum albumin and the blots were incubated overnight at 4°C with primary antibodies against AKT, pAKT, PI3K, pPI3K, mTOR, pmTOR, P70S6K, pP70S6K, Bax, Bcl-2, Cleaved Caspase 3, Cleaved PARP, Cleaved Caspase 8, Snail, E-cadherin, N-cadherin, Vimentin, Slug, Notch 1 & 2, Zeb, PTCH1, PTCH2, Gli1, SUFU, Shh and β-actin. Appropriate horseradish peroxidase-coupled secondary antibodies were used to detect the primary antibodies and visualized using enhanced chemiluminescence.

### Apoptosis by annexin V-FITC

Apoptosis was measured using Annexin V-FITC Apoptosis Detection Kit I. According to the manufacturer's instructions, HPAC, MIAPaCa-2, and PANC-1 cells were seeded in 6 well plates at a density of 2.5 × 10^5^ cells/well and treated with 25μM gedunin for 24h. Pancreatic cell death was analyzed using a FACS Accuri C6 flow cytometer (San Jose, CA, USA).

### TUNEL assay

DeadEnd Fluorometric TUNEL assay was conducted to analyze DNA fragmentation in cells undergoing apoptosis. Pancreatic cancer cells were seeded in 8-well chambers at a density of 5 × 10^3^ cells and treatment with 25μM gedunin for 24h (25412610). After fixing with 4% formaldehyde, the cells were washed with PBS and permeabilized using 0.2% Triton X-100. Following equilibration, cells were labeled using TdT reaction mix and incubated for 60 min at 37°C in a humidified chamber. Subsequently, 2X SSC were added for 15 min to stop the reaction. Apoptotic cells were detected using Nikon laser scanning confocal microscope [42].

### Confocal microscopy analysis

HPAC cells (1 × 10^4^ cells/well) were seeded in 8-well chamber slides and treated after 24h with 25μM gedunin, 1μg/ml rhShh, 5μM GANT-61, 1μg/ml rhShh and 25μM gedunin, 5μM GANT-61 and 1μg/ml rhShh. Then 24h after incubation with the respective treatments the cells were fixed with 100% methanol and 100% acetone (ratio 1:1). Membrane permeabilization was done using 0.2% Triton X in PBS for 20 min and blocked with 5% BSA. Slides were then incubated with Shh (sc-9024) and Gli1 (sc-20687) antibodies (Santa Cruz Biotechnology, Santa Cruz, CA, USA) for 24h followed by Alexa fluor 488-conjugated anti–rabbit secondary antibody (Life Technologies, Grand Island, NY, USA). Images were captured by Nikon laser scanning confocal microscope.

### Xenograft study

All the animal experiments performed were approved by the Texas Tech University Health Sciences Center Institutional Animal Care and Use Committee. Female 4–6 week old athymic nude mice were procured from Harlan Laboratories (Madison, WI, USA). HPAC cells were implanted subcutaneously in the right and left flanks (1 × 10^6^ cells/flank) of each animal (n=6). Once palpable tumors were formed (~100 mm^3^), gedunin (20 mg/kg body weight; 5 days/ week for 1 month) was administered via intraperitoneal injections to the treatment group, control animals received vehicle (Dimethyl Sulfoxide) treatment. All animals were regularly monitored and both tumor volume and body weight were recorded weekly. Tumor volume was calculated and compared between control and treated groups using the formula 4/3π×r_1_^2^×r_2_, where r_1_ is the minor radius and r_2_ is the major radius. After one month of treatment the mice were euthanized and the tumors, brain, lung, and liver tissues were surgically excised. A portion of the tissues were fixed in 10% formalin for histopathological analysis and immunohistochemistry while the rest of the tissue was snap frozen in liquid nitrogen and used for molecular analysis.

### Hematoxylin and eosin staining

Formalin fixed paraffin embedded tissues were deparaffanized, gradually dehydrated, and stained with H&E staining. Subsequently, brain, liver and lung tissues from both control and gedunin–treated mice were evaluated for micrometastatic abrasions. Images were obtained using Nikon Microscope– ECLIPSE 50i at 40X magnification.

### IHC

Formalin–fixed paraffin–embedded tumors were deparaffanized in xylene and rehydrated via decreasing ratios of ethanol baths. After antigen retrieval, tissue samples were blocked and incubated with antibodies against Shh and Gli1. Antibody binding was detected using ultra Marque polyscan HRP labeling (Cell Marque, Rocklin, CA, USA). Tissue slides were stained with DAB chromogen and counterstained with hematoxylin followed by dehydration through increasing ethanol dilutions ending in a xylene bath. After slides were sealed with mounting media (Surgipath Medical Industries, Richmond, IL, USA), images were captured using a Nikon Microscope–ECLIPSE 50i at 40X magnification.

### Statistical analysis

Statistical analysis were performed using Graphpad Prism version 5.03 software (La Jolla, CA, USA). The repeated measures analysis of variance was used to evaluate the dose and time response to gedunin treatment. Multiple comparisons between groups with significant differences were analyzed using Dunnett post–hoc test. Paired t–test was done to analyze intergroup differences. The values of p <0.05 were considered as statistically significant.
